# The Role of Nutraceuticals and Functional Foods in Mitigating Cellular Senescence and Its Related Aspects: A Key Strategy for Delaying or Preventing Aging and Neurodegenerative Disorders

**DOI:** 10.3390/nu17111837

**Published:** 2025-05-28

**Authors:** Sara Ristori, Gianmarco Bertoni, Elisa Bientinesi, Daniela Monti

**Affiliations:** Department of Experimental and Clinical Biomedical Sciences “Mario Serio”, University of Florence, 50134 Florence, Italy; sara.ristori@unifi.it (S.R.); gianmarco.bertoni@unifi.it (G.B.); elisa.bientinesi@unifi.it (E.B.)

**Keywords:** cellular senescence, inflammaging, healthy aging, neurodegenerative diseases, nutraceuticals, functional foods, Alzheimer’s disease, Parkinson’s disease

## Abstract

As life expectancy continues to increase, it becomes increasingly important to extend healthspan by targeting mechanisms associated with aging. Cellular senescence is recognized as a significant contributor to aging and neurodegenerative disorders. This review examines the emerging role of nutraceuticals and functional foods as potential modulators of cellular senescence, which may, in turn, influence the development of neurodegenerative diseases. An analysis of experimental studies indicates that bioactive compounds, including polyphenols, vitamins, and spices, possess substantial antioxidants, anti-inflammatory and epigenetic properties. These nutritional senotherapeutic agents effectively scavenge reactive oxygen species, modulate gene expression, and decrease the secretion of senescence-associated secretory phenotype factors, minimizing cellular damage. Nutraceuticals can enhance mitochondrial function, reduce oxidative stress, and regulate inflammation, key factors in aging and diseases like Alzheimer’s and Parkinson’s. Furthermore, studies reveal that specific bioactive compounds can reduce senescence markers in cellular models, while others exhibit senostatic and senolytic properties, both directly and indirectly. Diets enriched with these nutraceuticals, such as the Mediterranean diet, have been correlated with improved brain health and the deceleration of aging. Despite these promising outcomes, direct evidence linking these compounds to reducing senescent cell numbers remains limited, highlighting the necessity for further inquiry. This review presents compelling arguments for the potential of nutraceuticals and functional foods to promote longevity and counteract neurodegeneration by exploring their molecular mechanisms. The emerging relationship between dietary bioactive compounds and cellular senescence sets the stage for future research to develop effective preventive and therapeutic strategies for age-related diseases.

## 1. Introduction and Background

Life expectancy has dramatically increased in nearly all nations, and the global population has tripled since the mid-twentieth century. By 2030, the global human population is projected to grow to approximately 8.5 billion, with an additional 1.18 billion people expected in the following two decades, reaching 9.7 billion in 2050 [1]. Aging is rapidly accelerating worldwide. By 2050, the number of people over 65 is expected to more than double, reaching 1.5 billion, representing 16% of the global population. While this trend is more intense in developed countries—26% of the European and North American population are over 65—it has also become a significant global phenomenon that affects developing countries [2,3]. Nevertheless, insufficient evidence suggests that an increase in longevity correlates with a more extended period of good health [4]. Indeed, a notable difference exists between lifespan, defined as the total years lived, and healthspan, which refers to the duration without disease [5]. Extending lifespan without postponing the onset of diseases or lessening their severity would worsen the healthspan–lifespan gap. Advanced age is marked by the emergence of various complex health conditions, such as age-related diseases (ARDs) and geriatric syndromes (GSs), also referred to as “chronic or non-communicable” diseases, which are the leading cause of mortality and disability worldwide [6].

**Figure 1:**
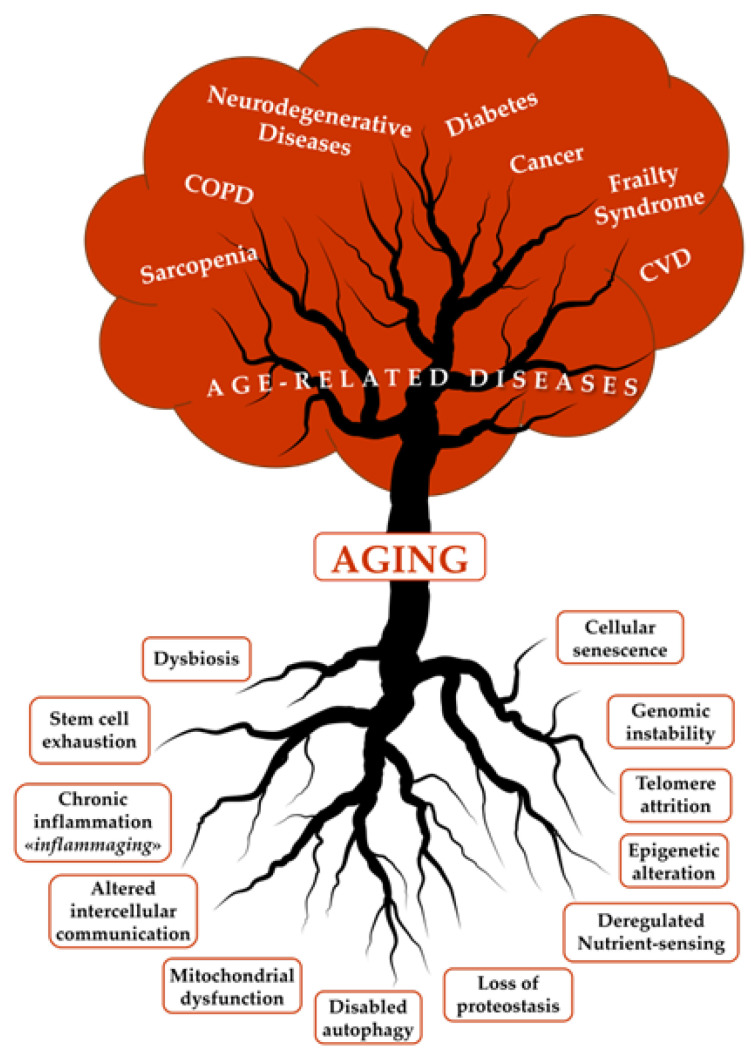
The hallmarks of aging. Aging is a multifactorial process at various levels, from molecules to cells, organs to systems, ultimately affecting the entire organism. In 2023, López-Otín and colleagues refined the framework of aging hallmarks, identifying 12 key features: dysbiosis, stem cell exhaustion, chronic inflammation (*inflammaging*), altered intercellular communication, mitochondrial dysfunction, epigenetic alterations, impaired macroautophagy, loss of proteostasis, deregulated nutrient sensing, telomere attrition, genomic instability, and cellular senescence [12].

Aging is an inescapable, natural, and universal feature of most living organisms that results from environmental, genetic, epigenetic, and stochastic factors, each contributing to the overall phenotype [7,8]. As humans age, damaging changes accumulate in the molecules, cells, and tissues, leading to a decline in normal physiological functions and a reduced ability to maintain adequate homeostasis. The increased susceptibility to various stressors and reduced ability to adapt to the environment lead to clinical diseases, where genetic, epigenetic, and environmental factors play a key role [9]. Geroscience provides a new perspective on gerontology by investigating the link between aging and ARDs. Both epidemiological evidence and experimental research demonstrate that aging is the principal risk factor for ARDs and GSs. Geroscience posits that aging and ARDs/GSs share a fundamental set of biological mechanisms, and twelve biological processes have been identified as the critical pillars of aging and ARDs (Figure 1). The hallmarks of aging appear to be closely interconnected, forming a finely controlled network; cellular senescence and inflammation represent the “umbrella” that encompasses all these mechanisms [10,11].

These hallmarks are intricately linked and interconnected and represent the fundamental changes associated with aging (the roots of aging). As aging advances, it broadly supports the onset of ARDs, including chronic obstructive pulmonary disease (COPD), sarcopenia, diabetes, cancer, frailty syndrome, cardiovascular diseases (CVDs), and neurodegenerative disorders like Alzheimer’s and Parkinson’s diseases. Just as a tree derives nourishment from its roots, these health issues represent the fundamental biological alterations of aging.

All hallmarks are time-dependent on the aging process and can be manipulated by laboratory experiments to accelerate—or by therapeutic interventions to slow down—the aging process [12]. Therefore, medicine’s primary objective should be to tackle the aging process and enhance the mechanisms that can prevent, delay, or counteract ARDs/GSs [13,14]. An integrated hypothesis proposes that ARDs/GSs manifest an accelerated aging process, indicating that the aging phenotype and ARDs/GSs are not distinct entities, but the outcomes of the same common set of molecular and cellular processes, likely occurring at varying rates [13]. Which determinants make aging trajectories more or less steep? Environmental conditions, such as the intensity and types of stressors, as well as lifestyle, are important health factors. However, the body’s ability to respond to and adapt to these stressors is even more crucial. This capacity is influenced at least partly by an individual’s genetic background and epigenetic changes, which play a significant role in various adaptation and remodeling processes.

Hormesis is a potential mechanism that explains the relationship between healthy aging and the development of ARDs/GSs. Hormesis refers to the beneficial effects of cellular responses to mild, repeated stress [15,16]. This theory suggests that regular exposure to mild stressors can positively impact various organs and systems, including adipose tissue, the liver, the brain, and the immune system [15], ultimately leading to enhanced overall health. Lifelong low-intensity stressors activate maintenance and repair mechanisms that positively affect health. However, increasing the intensity of these stressors can surpass the ability of organs and systems to adapt, resulting in detrimental effects. The emerging concept defines aging as malleable. By targeting the hallmarks of biological aging, such as cell senescence and its interdependent features, it is possible to alleviate ARDs and dysfunctions, thereby extending longevity. Additionally, using external molecules to boost the body’s natural cellular defense mechanisms is proposed as a promising anti-aging strategy centered on hormetic-based protection [17].

A recent multi-omics data study has shown that different organs and tissues can age at distinct rates within the same individual [18]. Brain pathologies and changes in brain structure are commonly seen in aging [19], with significant modifications in the brain’s intricate microstructure resulting in cognitive decline [20]. Brain morphology evolves with age and most commonly undergoes significant atrophy [21]. These changes are associated with, if not directly the cause of, cognitive deficits such as memory loss [22,23], reduced motor performance [24], and alterations in behavior [25].

Among neurodegenerative diseases, Parkinson’s disease (PD) and Alzheimer’s disease (AD) are the most common. Usually, they have a late debut of manifestation with a subsequent stage of progression leading to signs of dementia, with similar symptoms, such as memory impairment, orientation problems, and difficulties in performing service functions. In central nervous system (CNS) health, the brain aging trajectory is closely linked to cellular damage accumulation and the onset of neurodegenerative processes. An emerging pivotal factor contributing to the decline in brain structure and function is cellular senescence, a state of stable growth arrest, macromolecular damage, and altered metabolism associated with a hypersecretory and pro-inflammatory phenotype known as the senescence-associated secretory phenotype (SASP). Neuroinflammation may be one of the factors responsible for increased cognitive decline and the risk of AD and PD [26].

This article comprehensively reviews recent advancements concerning the impact of various nutraceuticals and foods on cellular senescence and its interconnected aspects. It delves into key factors associated with this process, such as inflammation, macromolecular damage, mitochondrial dysfunction, and oxidative stress. These factors are critical as they represent common pathways linked to aging and neuronal damage. The review highlights how these dietary components may influence the above-mentioned mechanisms, potentially offering therapeutic avenues to mitigate the effects of aging at the cellular level.

A narrative search was conducted across multiple databases, including PubMed, Scopus, Web of Science, and Google Scholar, to gather the relevant literature for this review. The search utilized the following keyword combinations: “Antioxidant vitamins OR Polyphenols OR Spices OR Dietary Fibers OR Probiotics OR Prebiotics OR PUFAS OR Diets OR Mediterranean Diet OR Caloric Restriction AND Aging AND Cellular Senescence OR Neurodegeneration OR Alzheimer’s disease OR Parkinson’s disease”. The search included only articles published in English and those available via open access to ensure the inclusion of the most recent advancements. Studies included in the review were required to focus on preclinical (in vitro and in vivo experiments) and clinical studies, specifically addressing how nutraceuticals influence the mechanisms of cellular senescence in aging and neurodegenerative diseases. Additionally, the review cites papers considered pioneering in the field.

## 2. The Role of Senescence in Aging and Neurodegenerative Diseases

Senescence is considered a highly dynamic, multistep process over which the properties of senescent cells continuously evolve and diversify context-dependently [27]. Formally described in 1961 by Hayflick and colleagues, cellular senescence was initially observed in normal human fibroblasts that stopped proliferating after a finite number of divisions [28]. Subsequent studies have proven that a variety of stressors, including oxidative stress, DNA damage, oncogene activation, mitochondria deterioration, chemotherapy, and exposure to ionizing radiation (IR), can trigger “stress-induced premature senescence” in vitro [29,30].

**Figure 2:**
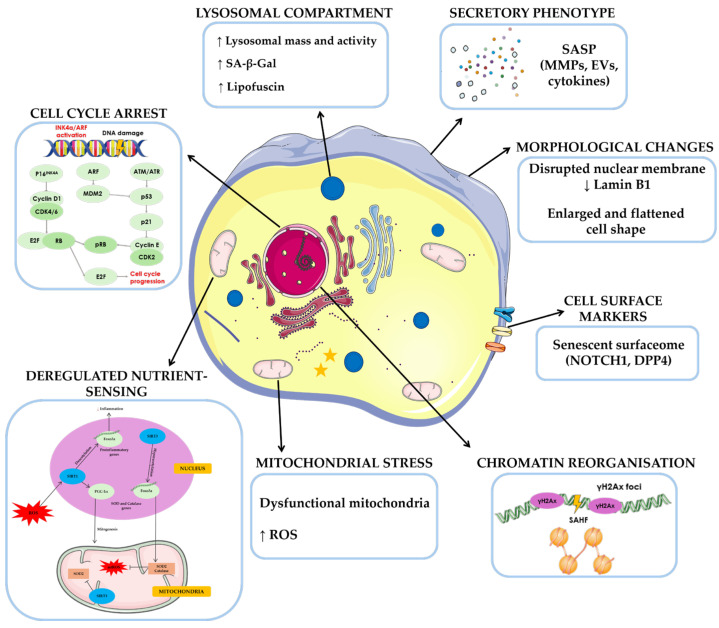
The hallmarks of cellular senescence. Senescent cells undergo distinct morphological and molecular alterations defining cellular senescence’s hallmarks. At the genomic level, senescence is characterized by stable cell-cycle arrest, primarily driven by the p16^INK4a^/Rb and p21^CIP1^/p53 pathways. Chromatin reorganization is a key feature, marked by senescence-associated heterochromatic foci (SAHFs) and γ-H2AX foci, indicating DNA damage. Lysosomal alterations are also evident, with increased lysosomal mass and activity, elevated expression of senescence-associated β-galactosidase (SA-β-Gal), and lipofuscin accumulation. Senescent cells exhibit notable morphological changes, including enlargement, flattening, and modifications in membrane structure. A feature of nuclear dysfunction is the downregulation of Lamin B1, leading to nuclear envelope instability. The senescent surfaceome is enriched with specific channels and receptors, such as NOTCH1, DPP4, and B2M. Mitochondrial dysfunction is another critical feature, leading to increased ROS production and deregulated nutrient sensing. SIRT1 and SIRT3 play pivotal roles in mitochondrial homeostasis and oxidative stress regulation. SIRT1, located in the nucleus, modulates mitochondrial function by deacetylating FOXO3a, reducing inflammatory protein expression, and activating PGC-1α to promote mitochondrial biogenesis. Oxidative stress inactivates SIRT3, leading to SOD2 hyperacetylation and increased mitochondrial ROS (mtROS), creating a vicious cycle of oxidative stress and mitochondrial dysfunction. SIRT3-mediated deacetylation of FOXO3a and SOD2 counteracts ROS accumulation by upregulating antioxidant defenses, including catalase. Finally, a defining feature of senescence is the SASP, characterized by the secretion of pro-inflammatory cytokines (e.g., IL-1β, IL-6, TNF-α, etc.) and matrix metalloproteinases (MMPs), often encapsulated in extracellular vesicles (EVs). ↑ increase; ↓ decrease.

Senescence activation leads to several molecular changes and distinct phenotypic alterations, including chromatin remodeling, shortened telomeres, the accumulation of DNA damage and reactive oxygen species (ROS), the activation of cell-cycle inhibitory pathways, lysosome enlargement, macromolecular disruption, metabolic disbalance, apoptosis resistance, and the SASP [31]. The SASP is characterized by the synthesis of various biologically active molecules, such as inflammatory mediators, growth factors, and extracellular matrix proteins. These factors reinforce the senescent phenotype through autocrine or paracrine signaling, and can also affect the microenvironment, influencing neighboring cells and distant locations within the organism [30] (Figure 2).

As the number of senescent cells increases with age, there is increasing evidence suggesting their involvement in the pathogenesis of ARDs [32,33,34], including neurodegenerative diseases such as AD and PD [35].

Moreover, PD and AD are called “protein-misfolding diseases” because deposits of improperly folded and modified proteins are detected in specific areas of the patient’s brain, leading to neuronal damage [36]. It has been reported that the final dysfunction and neuronal loss observed in neurodegenerative diseases are often accompanied by malfunctions of other types of CNS cells, such as microglia and astrocytes. Various types of cells in the nervous system have been identified as undergoing the senescence process, including neural stem cells, neurons, astrocytes, oligodendrocytes, and microglia. In a state of senescence, microglia are neurotoxic and become detrimental in many neurodegenerative diseases by producing inflammatory cytokines, superoxide anions, and nitric oxide, promoting the phenomenon of *“oxi-inflamm-aging”*, which contributes to neuropathogenesis [37,38,39].

Evidence shows that senescent astrocytes accumulate in AD and PD patients, promoting inflammation through the SASP factors [40,41,42]. Indeed, several SASP factors, including MMP-3, IL-1α, IL-6, and IL-8, are increased in PD and AD brains, indicating that cellular senescence could contribute to neurodegeneration [35,43,44]. In addition, in the brain tissue of PD patients, α-synuclein deposition correlates with increased senescent cell accumulation and higher SA-β-Gal expression, suggesting the role of cellular senescence in the pathogenesis of PD [44]. Conversely, the attenuation or elimination of cellular senescence has been shown to alleviate neuroinflammation in AD and PD models [43,45]. Moreover, a recent study revealed that senescent neurons with tau neuropathology are prevalent in patients with AD [46], while the removal of accumulated senescent glial cells attenuated cognitive decline and age-related neurogenerative disorders [47].

Therefore, eliminating senescent cells within the CNS, or at least delaying their senescence, and mitigating the adverse effects of a spreading SASP have been identified as targets for the prophylaxis and adjunctive treatment of neurodegenerative diseases.

Furthermore, the SASP can be viewed as an “inflammatory chain reaction” that promotes damaging effects and contributes to systemic inflammaging; thus, biomolecules with antioxidants and anti-inflammatory properties would be beneficial not only as protectors against senescence induction, but also as tools to extinguish the inflammatory ripple effect [39].

## 3. Nutritional Interventions to Slow Down Aging

The hallmarks of aging constitute an interconnected network of fundamental mechanisms that influence aging and can be modulated by lifestyle factors, including nutrition, to improve human healthspan [48]. Aging is a malleable process characterized by an intra- and inter-individual heterogeneous and dynamic balance between accumulating damage and repair mechanisms. Nutritional interventions that help slow this process can reduce cellular damage and the accumulation of senescent cells or enhance the ability of cells, tissues, or the organism to repair or adapt to this damage [49]. In this context, several natural compounds, known as “bioactive compounds”, can interact with biological processes, and when present in food, they are referred to as “nutraceuticals” [50]. As discussed in the following paragraph, many studies focus on identifying bioactive compounds with preventive effects against pathological conditions or with broader anti-aging properties. Moreover, emerging evidence suggests that dietary factors can influence brain health and cognitive function, providing a promising avenue for intervention [51].

In this context, it is also important to highlight that some nutraceuticals may exhibit hormetic behavior, displaying a biphasic dose-response relationship in which low doses provide beneficial effects, whereas high doses may be detrimental.

These positive effects at low concentrations arise from stimulating adaptive stress responses, ultimately enhancing the body’s resilience to various stressors. Recent findings show that several natural compounds may act in a hormetic-like manner. These hormetic compounds may mediate health-promoting actions by triggering one or more adaptive stress response pathways [52]. This phenomenon is particularly evident among polyphenols, such as curcumin and resveratrol [53,54]. Interestingly, combining different nutraceuticals, such as probiotics and polyphenols, a hormetic nutritional approach, exerts potent neuroprotective and therapeutic effects by activating antioxidant Nrf2 signaling pathways. Consequently, these hormetic nutrients may prevent and treat inflammation-driven pathophysiological changes in gut microbiota diversity that contribute to nervous system disorders via the gut-brain axis [54]. Reading the following reviews can comprehensively understand the topic [55,56,57].

Recent studies have focused on discovering nutraceuticals that mimic the effects of metformin and rapamycin, inhibiting mTOR, without their side effects. Researchers have individuated withaferin A, allantoin, ginsenoside, and epigallocatechin gallate as promising candidates for experimental validation [58,59]. These substances induced strong activation of the cAMP pathway, which was recently found to induce anti-aging effects similar to caloric restriction (CR) via the up-regulation of sirtuins (SIRTs) [60]. SIRTs, particularly SIRT1 and SIRT3, are key regulators of cellular metabolism, stress responses, and aging. As NAD+-dependent deacetylases, they are activated under CR, promoting longevity and healthspan by modulating energy metabolism, mitochondrial function, and stress resistance. SIRT1 acts as a nutrient sensor, regulating epigenetic modifications, mitochondrial quality, and anti-inflammatory responses, while SIRT3 enhances mitochondrial protein deacetylation, optimizing oxidative metabolism and aerobic fitness, both contributing to the lifespan-extending effects of CR [61,62,63].

CR consists of a 25–50% calorie reduction compared to a standard diet, with preservation of vitamin and mineral supply [64]. In addition to SIRTs, CR modulates other key nutrient signaling pathways, including insulin/IGF-1, mTOR, and AMPK, leading to a reduction in oxidative stress, enhancement of mitochondrial function, activation of anti-inflammatory responses, stimulation of neurogenesis, and increased synaptic plasticity, emphasizing the positive impact of CR on brain functions. These effects can delay cellular senescence and may significantly mitigate age-related functional decline [65]. Experimental studies have reported that CR reduces molecular features of cellular senescence in different human and mouse models [33,66,67]. Interestingly, a recent study demonstrated that moderate CR could decrease circulating biomarkers of cellular senescence in healthy young-to-middle-aged humans without obesity, highlighting the impact of lifestyle [68]. Moreover, dietary restriction and plant-based dietary patterns have been linked to improved key clinical outcomes related to aging, particularly body composition changes, lipid profile, blood pressure, lipid peroxidation, inflammation, and cardiometabolic risk [66,69,70,71,72,73,74,75]. Despite the mechanisms not being fully elucidated, these benefits suggest that such dietary approaches may be crucial in promoting healthy aging by modulating metabolic and inflammatory pathways central to age-related physiological changes and disease prevention. However, in CR, the timing of initiation is a critical factor; when started at an early age, it is associated with beneficial effects [76]. Conversely, in older adults, CR may exacerbate sarcopenia and osteopenia, contributing to muscle and bone loss [77].

In addition to CR, other dietary patterns have been proposed to promote healthy aging with hormetic behavior [78,79]. Among these, the Mediterranean diet (MedDiet) is the most studied. The MedDiet is characterized by a high intake of vegetables, fruits, whole grains, and fish, and it has demonstrated significant health benefits, including the prevention of ARDs. Its protective effects are mainly attributed to its rich composition of bioactive compounds that help modulate oxidative stress, inflammation, and metabolic processes, further supporting its role in longevity and overall well-being [80,81,82,83,84] and reducing cognitive impairment [85]. Intriguingly, emerging proofs suggest that adherence to the MedDiet may contribute to delaying cellular senescence [86]. In older adults, adherence to the MedDiet has been associated with a lower proportion of endothelial cells with shorter telomeres, an effect likely mediated by decreased ROS production and apoptosis [87]. Similarly, Mantilla-Escalante suggests that long-term adherence (1 year) to the Med-Diet, particularly when enriched with nuts, can modulate the expression of several mi-croRNAs (miRNAs) involved in cellular senescence, including cell-cycle regulators and pro-inflammatory markers. The MedDiet, through miRNA-mediated gene modulation, may influence fundamental mechanisms of aging and cellular homeostasis [88].

Even if the mechanisms through which food influences aging are not fully understood, several bioactive compounds have been reported to function as epigenetic modulators, influencing gene expression, chromatin organization, DNA methylation patterns, and non-coding RNA expression [89,90].

Interestingly, the human epigenome is influenced by exogenous factors such as nutrition, a field explored in nutritional genomics. Both the quality and quantity of diet have been found to modulate DNA methylation and mental health epigenetically [91].

Additionally, an intriguing hypothesis suggests that bioactive compounds in food may extend healthspan by modulating the SASP, indicating new strategies to slow the onset and progression of ARDs [92]. Since the anti-aging effects of natural compounds have only recently begun to be scientifically evaluated, very few notions are available about their properties and ability to exert anti-SASP and/or senolytic activity. However, nutrition is often considered one of the most promising modifiable risk factors for ARDs, including neurodegenerative diseases, a contention fully appreciated in multidomain intervention studies [93,94,95].

While all-natural foods are inherently functional due to their composition, the concept of functional foods emerged from the observation that certain manufactured foods, enhanced with additional ingredients, can further improve human health [96]. This category includes conventional foods enriched with bioactive compounds such as vitamins, minerals, and phytochemicals [91,92], directly impacting nutritional health by enhancing overall well-being or reducing disease risk [97]. Among the various nutraceutical-enriched foods, olive oil, milk, and yoghurt stand out for their potential health benefits. Extra virgin olive oil has been extensively studied for its positive effects on telomere length, diabetes, cognitive function, and various hallmarks of aging, including cellular senescence [98]. Martucci et al. studied, through an interventional trial with 48 elderly volunteers, the impact of fortified milk on inflammaging parameters. The fortified milk was enriched with omega-3 fatty acids (EPA, DHA), various vitamins, and trace elements, finding improved levels of micronutrients and the omega-3 index, along with reduced arachidonic acid (AA), homocysteine, and omega-6/omega-3 ratios [99]. Yoghurt, rich in anti-inflammatory and B-vitamin content, may help protect against cognitive decline. Tillisch et al. showed in a randomized trial on healthy women that a four-week intake of fermented milk affected brain function changes [100].

Functional foods play a crucial role in healthy aging by addressing factors like oxidative stress, inflammation, and mitochondrial dysfunction. Their positive effects on aging mechanisms suggest potential benefits for aged people [101].

Due to modulating many biological mechanisms in mammalian bodies and cells, the following anti-aging mechanisms of functional foods could be proposed: (i) stabilizers of mitochondrial membranes and enhancers of mitochondrial function—agents that avoid cell death by apoptosis or necrosis; (ii) metal-chelating activities; (iii) antioxidants; (iv) inducers of apoptosis of preneoplastic and neoplastic cells [102,103,104,105].

The distinction between nutraceuticals and functional foods is often blurred due to their intrinsic overlap, as nutraceuticals represent a specific subset of functional foods. Given this complexity, our review will specifically focus on nutraceuticals to provide a more structured and comprehensive analysis of their role in neurodegenerative diseases, specifically AD and PD. By narrowing our scope, we aim to offer a clearer perspective on their mechanisms of action and potential therapeutic applications.

## 4. Nutraceutical Interventions in Neurodegenerative Disorders: Focus on Parkinson’s and Alzheimer’s Diseases

The aging brain is highly susceptible to neurodegenerative diseases, but the exact mechanisms through which senescence in the CNS contributes to neuropathogenesis remain unclear. The number of senescent cells increases with age, and there is growing evidence suggesting the involvement of cellular senescence in the neuropathogenesis of AD and PD, resulting in a significant increase in chronic neuroinflammation due to the SASP [106]. Therefore, countering and removing senescent cells in the brain, or at least postponing their senescence and alleviating the adverse effects of a spreading SASP, could be a strategy for helping to slow the progression of AD and PD or delaying their onset.

This section reviews studies investigating nutraceutical compounds that may mitigate cellular senescence processes in the brain, including neuroinflammation and the reduced expression of anti-apoptotic proteins such as Bcl-2 and Bcl-xl, as well as compounds that demonstrate senostatic and senolytic effects. Although direct evidence linking nutraceuticals to cellular senescence in neurodegenerative diseases is currently limited, this field has considerable potential. Various nutraceuticals have shown beneficial effects across numerous models by modulating traits associated with senescence, indicating that further research may provide valuable insights into their advantages.

Given the established role of senescent cells in neurodegenerative diseases, we suggest that nutraceutical compounds affecting senescence-associated features may yield beneficial outcomes in these conditions. However, due to the lack of direct evidence, our discussion will primarily focus on key molecular and cellular mechanisms related to senescence rather than directly indicating their effects on senescent cells in neurodegeneration.

The compounds discussed are categorized based on their natural origin. The results from studies on their effects as senotherapeutic substances in aging and neurodegenerative diseases are presented below and summarized in Table 1.

### 4.1. Antioxidant Vitamins

A major contributor to aging and ARDs, such as AD [191] and PD [192], is oxidative stress induced by free radicals. Oxidative stress can directly activate glial cells, mainly by priming astrocytes, resulting in their interaction with neurons and the subsequent release of immune mediators such as nitric oxide (NO), additional ROS, pro-inflammatory cytokines, and chemokines. These mediators act as neurotoxins, propagating inflammation within the CNS [193].

Accumulating evidence from mouse models of accelerated senescence indicates that ascorbic acid (AAC) plays a rescuing role in premature aging. Moreover, although the precise role of AAC in the CNS remains partially understood, studies have demonstrated that its concentration in the cerebrospinal fluid (200–400 mM) far exceeds that found in cerebral parenchyma and plasma (30–60 nM) [194]. Overall, AAC exhibits notable nootropic properties [195] and has been shown to decrease acetylcholinesterase activity in mice [107]. In addition, it facilitates the differentiation of neuronal and astrocyte precursors, thereby promoting synaptic maturation [196]. AAC is also essential for the biosynthesis of catecholamines, peptide amination, myelin formation, and the enhancement of synaptic function, while providing neuroprotection against glutamate toxicity [197,198].

In PD, dopamine metabolism generates oxidative stress products that contribute to accumulating abnormal proteins that are characteristic of PD [199]. Current therapeutic strategies for PD primarily alleviate symptoms, but they do not halt disease progression, rendering treatment particularly challenging.

Although early studies indicated that AAC supplementation could mitigate oxidative damage in in vitro and animal models [108,109], more recent investigations have yielded inconsistent results [200]. Notably, AAC levels are lower in the substantia nigra compared to other brain regions [201,202], heightening its vulnerability to oxidative stress [203]. Furthermore, AAC has been shown to enhance the production of dihydroxyphenylalanine (DOPA); Seitz et al. observed a dose-dependent overproduction of DOPA in the human neuroblastoma cell line SK-N-SH following incubation with AAC (100–500 mM) for 2 h [204]. Nonetheless, AAC has been demonstrated to improve the absorption of levodopa in elderly PD patients with poor levodopa bioavailability, thereby enhancing its therapeutic efficacy and reducing its side effects [205,206]. Moreover, AAC is critical for brain development; one study reported that AAC treatment induced a tenfold increase in dopaminergic differentiation in CNS precursor cells derived from E12 rat mesencephalon [207]. In vivo, a cohort study of 1,036 PD patients further supported the neuroprotective role of AAC, demonstrating that higher dietary intake was significantly associated with a reduced risk of PD [208], although some studies have not corroborated these findings [209,210].

In contrast, the neuroprotective effects of vitamin E are thought to arise from its ability to prevent oxidative stress and inhibit apoptosis. Vitamin E has been shown to reverse impaired synaptic plasticity in mouse models [114] and reduce ROS levels in Drosophila models [109]. Additional evidence underscoring the role of oxidative stress in PD includes observations that cellular antioxidants such as glutathione (GSH) are depleted in PD [211].

Specific isoforms of vitamin E, such as γ-tocotrienol and δ-tocotrienol, exert neuroprotective effects through the ERβ-PI3K/Akt signaling pathways in SH-SY5Y cells [115]. Moreover, δ-tocotrienol has been found to prevent dopaminergic neuron loss and improve motor function in mouse models of PD; its neuroprotective effect, however, was attenuated by ER inhibitors [116]. In an MPTP-induced PD model in C57/B1 mice, vitamin E-deficient animals were markedly more susceptible to MPTP toxicity, exhibiting increased lethality and greater depletion of dopamine metabolites in the substantia nigra [212]. Perry et al. [117] similarly reported that mice treated with daily subcutaneous injections of high-dose α-tocopherol (αT) (2350 mg/kg body weight) 48 h before and 72 h after MPTP administration experienced partial protection against the loss of striatal dopamine and dopaminergic neurons in the substantia nigra. In supporting these experimental findings, a cross-sectional study involving participants over 40 years of age found that higher vitamin E intake was significantly associated with a reduced risk of PD [118].

Conversely, L-AAC has also garnered attention for its beneficial effects on AD [213]. The primary neuroprotective mechanisms attributed to AAC include ROS-scavenging activity, neuroinflammation modulation, Aβ fibrillation inhibition, and the chelation of metals such as iron, copper, and zinc [214]. Furthermore, AAC has been shown to protect SH-SY5Y neuroblastoma cells from Aβ-mediated apoptosis [110] and, when administered orally, to reduce oxidative stress and neuroinflammation induced by Aβ fibrils in rats [111].

In contrast, vitamin E is a potent antioxidant that scavenges free radicals primarily through a hydrogen atom transfer mechanism [215]. Vitamin E plays a crucial role in the brain, is one of the most potent antioxidants, and has shown significant benefits in AD [216]. It counteracts Aβ-induced oxidative stress [119]; for instance, vitamin E has been demonstrated to prevent Aβ_1-42_-induced protein oxidation, ROS production, and neurotoxicity in primary rat embryonic hippocampal neuronal cultures [119]. Moreover, although Aβ_1-42_ reduces the surface expression of the principal glutamate transporter GLT-1 in adult mouse astrocytes, this detrimental effect is prevented by a water-soluble analogue of vitamin E [217]. Vitamin E also helps preserve calcium homeostasis and protects against damage from Aβ deposits near cell membranes [218]. Additionally, it can inhibit neuroinflammation by suppressing the production of prostaglandins E_2_ and D_2_, along with reducing cyclooxygenase and lipoxygenase activity [120,121].

Numerous research studies demonstrate that AAC and vitamin E can reduce cell senescence. However, limited evidence directly links cellular senescence, neurodegenerative diseases, and antioxidant vitamins. Most research has concentrated on other cell types, and only a few studies have investigated the potential role of antioxidant vitamins in influencing senescence and its related pathways in brain cells.

We highlight some relevant findings to offer a broader perspective on the capacity of antioxidant vitamins to modulate cellular senescence. Specifically, AAC downregulates SA-β-Gal and cell-cycle inhibitors (p53, p21, p16, and pRb) while upregulating activators (E2F1/2), reducing senescence in human dermal fibroblasts, hairless mice models, and LPS-treated human apical papilla cells [112,113].

Limited studies are also available regarding vitamin E supplementation. Vitamin E, including its phosphorylated form αTP, reduces SA-β-Gal activity in human fibroblasts and endothelial cells, with greater efficacy observed in cells from aged donors [122,123]. Specifically, vitamin E reduces SA-β-Gal levels in cells from both young and aged donors when reaching replicative senescence. This effect is also observed in earlier fibroblast passages from older subjects, likely due to a phosphorylated form of vitamin E, α-tocopheryl phosphate (αTP), which occurs in aging caused by reduced conversion to αT [123].

In summary, AAC exerts neuroprotective effects by scavenging ROS, modulating neuroinflammation, and supporting synaptic function, while vitamin E mitigates oxidative stress, preserves membrane integrity, and inhibits apoptosis. Although studies indicate potential positive outcomes on senescence-associated characteristics, findings remain inconsistent in AD and PD, highlighting the need for further research to elucidate their precise mechanisms and therapeutic potential.

In addition to the well-known antioxidant vitamins, several others exert indirect antioxidant effects that may offer potential benefits for AD and PD.

Vitamin A, primarily through its active metabolite all-trans-retinoic acid (RA), plays a critical role in the CNS and maintains higher brain functions in aging individuals [219]. Although not classified as a direct antioxidant, RA exhibits significant indirect antioxidant properties [220]. In vitro studies have shown that vitamin A and β-carotene can inhibit the oligomerization of Aβ_40-42_ peptides [124]. Mechanistically, the activation of retinoic acid receptor alpha (RARα) increases the expression of ADAM10/α-secretase, an enzyme that mitigates amyloid burden by cleaving APP in a non-amyloidogenic pathway without affecting β- or γ-secretase activity, as demonstrated in mouse cortical neurons [125]. However, high concentrations of retinol exposure (10 μM for 24 h) in SH-SY5Y cells increased Aβ levels and reduced cell viability, suggesting hormetic behavior with dose-dependent cytotoxic effects [126]. With aging, retinoid signaling remains essential for brain homeostasis; however, senescence-associated impairments can diminish vitamin A signaling efficacy [221].

RA signaling is implicated in PD neurogenesis and the differentiation of striatal neurons. The disruption of RAR/RXR pathways, as observed in transgenic RXR−/− and/or RAR−/− mice, has been shown to impair synaptic plasticity in the hippocampus and other brain regions, highlighting the critical role of vitamin A signaling in PD pathophysiology [127,222].

Although structurally and functionally heterogeneous, the B-vitamin group encompasses key antioxidant defense and neuroprotection cofactors. Vitamins B9 and B12 are essential for one-carbon metabolism, a biochemical network crucial for DNA synthesis, epigenetic regulation, and redox balance [223]. One-carbon metabolism is often disrupted in AD, and vitamin B9 supplementation has been shown to restore metabolic balance and enhance cognitive outcomes, as evidenced by improved Mini–Mental State Examination (MMSE) scores in patients receiving vitamin B9 and B12 [128,129].

Beyond cognitive effects, vitamin B9 has also been reported to exert anti-aging properties. In the senescence-accelerated mouse prone 8 (SAMP8) model and primary astrocyte cultures, vitamin B9 supplementation (0–40 μM) reduced age-associated apoptosis and mitigated telomere attrition in cortical regions [130]. This finding is particularly relevant given that telomere shortening, a hallmark of replicative senescence, is often driven by oxidative stress and inflammation.

Plasma B12 levels correlate positively with telomere length and mitochondrial DNA copy number, declining with cellular aging [224,225,226].

Furthermore, vitamin B12 deficiency has been associated with the induction of cellular senescence markers in astrocytes. Specifically, B12-deficient astrocytes exhibit increased SA-β-gal activity and the upregulation of cell-cycle inhibitors p16^INK4a^ and p21^CIP1^, indicating a senescent phenotype [131].

Although not classified as antioxidants, vitamins A, B9, and B12 exhibit promising neuroprotective and anti-senescent properties. Therefore, their inclusion in this context is warranted, as they may pave the way for future studies exploring novel micronutrient-based interventions targeting age-related neurodegeneration and cellular senescence.

### 4.2. Polyphenols, Terpenes, and Terpenoids

Dietary polyphenols exhibit robust neuroprotective effects that extend well beyond their well-known antioxidant and anti-inflammatory properties. Circumstantial evidence indicates that these compounds modulate intracellular signaling pathways, alter gene expression, and influence enzyme activities, all contributing to their therapeutic potential in neurodegenerative diseases [227,228].

A growing body of research demonstrates that polyphenols can modulate cellular senescence in many research studies and models. For example, in vitro, the chronic treatment of pre-senescent neonatal human dermal fibroblasts with oleuropein aglycone, a prominent polyphenol in extra-virgin olive oil, resulted in a significant reduction in senescent cell numbers, as evidenced by decreased SA-β-Gal activity and lower p16 protein expression [229]. Similarly, compounds such as apigenin, quercetin, kaempferol, and wogonin have been shown to suppress the secretion of SASP markers, including IL-6, IL-8, and IL-1β [230]. Recent studies by Bientinesi et al. revealed that quercetin can prevent doxorubicin-induced senescence in human fibroblasts [132,133]. Quercetin not only alleviates the deleterious effects of the SASP in both U2OS and normal cells, but also protects fibroblasts from ROS-mediated damage, evidenced by reductions in senescence-associated heterochromatin foci (SAHF), Lamin B1 loss, and NF-κB nuclear translocation. Moreover, quercetin exhibits senolytic activity, reducing autophagy while increasing endoplasmic reticulum stress, thereby underscoring its multifaceted role in combating cellular aging. Several benefits have also been demonstrated in human in vivo studies. For instance, Maurya et al. showed that in human red blood cells, these flavonoids reduce malondialdehyde (MDA) levels while increasing GSH and membrane sulfhydryl (-SH) group levels [134].

Moreover, polyphenols can also modulate senescence through a hormetic mechanism, as shown for resveratrol and curcumin [56,57].

Curcumin is well known for its antioxidant properties, which are mediated through the Keap1/Nrf2/ARE pathway. It exhibits dual characteristics: at high concentrations, curcumin can be cytotoxic to mammalian cells, while at subtoxic levels, it activates adaptive stress responses. This protective effect is evidenced by its ability to guard against glucose oxidase-mediated toxicity in astrocytes and aged Tg2576 mice with advanced amyloid accumulation [148,149]. Interestingly, curcumin can paradoxically stimulate ROS production at higher concentrations.

Similarly, resveratrol displays dose-dependent effects. It activates the SIRT1 and AMPK pathways, which enhance mitochondrial function and promote autophagy at low doses. In contrast, higher doses of resveratrol have been observed to induce oxidative damage in both in vivo and in vitro AD models [139,231].

In the context of PD, dietary polyphenols appear to have beneficial effects. Flavonoids, a major subgroup of polyphenols, protect neurons against oxidative stress, suppress neuroinflammation, and modulate key intracellular signaling pathways critical for neuronal survival. These pathways, including protein kinase and lipid kinase signaling cascades, alter the phosphorylation state of target proteins and influence gene expression [232].

Moreover, histochemical evaluations in 6-OHDA-treated mouse models of PD have shown that green tea (a variant of tea obtained with non-treated leaves of Camellia sinensis) polyphenols markedly reduce ROS levels, lipid peroxidation, and intracellular nitrite/nitrate concentrations [138,233].

Ginkgo biloba extract, containing flavonoids, organic acids, proanthocyanidins, and terpenoids such as ginkgolides A, B, C, M, J, and bilobalide, has been reported to protect against nigrostriatal dopaminergic neurotoxicity in MPTP-induced PD models, with observed reductions in lipid peroxidation and enhancements in the activities of key antioxidant enzymes, such as SOD, GPx, and GSH reductase [136]. Notably, Ginkgo biloba extract inhibited monoamine oxidase B (MAO-B) in vitro, reducing dopaminergic neuron degeneration [136,137].

Resveratrol, a nonflavonoid polyphenol found in grapes and berries, has shown promise in mitigating oxidative stress in a rat model of PD [234,235] while enhancing the number of dopaminergic neurons at the synapses through MAO suppression, in addition to preventing glutamate release [236,237,238].

Additionally, oleuropein and its derivatives have been demonstrated to inhibit ROS accumulation and prevent PD pathology. In vitro, oleuropein aglycone stabilizes α-synuclein monomers, thereby preventing pathological aggregation [140], and it also inhibits α-synuclein fibril elongation, reducing the cytotoxic effects of α-synuclein oligomers [141]. Furthermore, oleuropein activates redox-sensitive transcription factors such as Nrf2, which may enhance the intracellular antioxidant capacity and contribute to neuroprotection [239].

Beyond PD, dietary polyphenols have been shown to have several benefits in AD, mitigating pathological manifestations partly due to their ability to cross the blood–brain barrier [240,241]. Polyphenols reinforce endogenous antioxidant defenses and attenuate protein oxidation [242]. By sequestering reactive oxygen and nitrogen species, these compounds prevent the formation of toxic Aβ oligomers and modulate tau-protein hyperphosphorylation, thereby impeding the development of neurofibrillary tangles (NFTs) [243]. Additionally, polyphenols may help preserve neuronal integrity by interacting with transcription factors such as CREB and NF-κB [244].

Studies on AD transgenic mouse models (APP/PS1 model) and patients’ post-mortem brains have revealed a senescent phenotype in oligodendrocyte progenitor cells (OPCs) within the Aβ plaque environment. Notably, these cells were sensitive to clearance by the senolytic cocktail dasatinib plus quercetin (D+Q). The treatment removed senescent OPCs and ameliorated Aβ plaque-associated inflammation and cognitive deficits in AD mice [135]. Meanwhile, in PD, direct evidence of the beneficial effects of D+Q has not been observed, even though some advantages have been shown in aging killifish [245].

Additionally, fisetin, a natural senolytic, has been shown to improve cognitive function in mouse models of AD and dementia [142]. Among the senolytics tested in multiple preclinical studies and increasing clinical trials, fisetin and D+Q appear to be the most effective [246,247].

Animal studies further substantiate the neuroprotective potential of polyphenols. For instance, mice receiving pomegranate juice, rich in polyphenols, exhibited significant improvements in both cued and spatial learning tasks, along with reduced hippocampal plaque loads, including both soluble and fibrillar forms of Aβ, as well as lower soluble Aβ_1-42_ levels [248]. Red wine polyphenols have been shown to interfere with Aβ oligomerization, thereby attenuating Aβ neuropathology and cognitive decline in Tg2576 mice [249]. Mori et al. [250] demonstrated that tannic acid shifts amyloid precursor protein metabolism toward a non-amyloidogenic pathway by lowering β-secretase 1 (BACE1) expression and β-secretase activity, decreasing Aβ peptide levels.

Similarly, grape-derived polyphenolics from Vitis vinifera grape seeds significantly inhibited Aβ aggregation in vitro and ameliorated cognitive deterioration in Tg2576 mice when administered orally [251].

Collectively, these findings illustrate the multifaceted neuroprotective potential of dietary polyphenols. By modulating intracellular signaling pathways, gene expression, and enzyme activities, polyphenols offer promising therapeutic avenues for preventing and treating neurodegenerative diseases, highlighting their potential as valuable agents in mitigating age-related cognitive decline and neuronal dysfunction.

In addition to polyphenols, terpenes and terpenoids exhibit notable neuroprotective, antioxidant, and anti-inflammatory properties, which may play a role in their anti-senescence effects. Among these compounds, limonene has shown hormetic-like activity.

While high doses of limonene are toxic to the Mediterranean fruit fly (Ceratitis capitata), with lethal doses recorded at 39.74 nL per male and 75.51 nL per female, lower doses (3.47 nL per male and 12.26 nL per female) have been found to extend lifespan. This highlights its potential in modulating aging processes [143].

Similarly, Ginsenoside F1, a minor saponin derived from Panax ginseng leaves, has been reported to suppress the SASP in astrocytes exposed to D-galactose. This effect is mediated by inhibiting the p38MAPK-dependent NF-κB signaling pathway, suggesting a potential role in reducing astrocyte-driven inflammation in AD [144]. Additionally, ginsenosides from P. ginseng have shown inhibitory activity against BACE1 activity in vitro, an important enzyme involved in Aβ production [145]. Artemisinin, a sesquiterpene lactone extracted from Artemisia annua, has shown moderate inhibition of acetylcholinesterase (AChE) at 1 mg/mL in vitro, alongside its known anti-inflammatory properties [146].

Another promising compound is Astragaloside IV (AS-IV), an antioxidant saponin extracted from the traditional Chinese medicinal herb Astragalus membranaceus Bunge. AS-IV exerts anti-inflammatory, neuroprotective, and longevity-promoting effects. In both replicative senescence (long-term culture-induced) and premature senescence models induced by LPS or MPP+, AS-IV attenuated astrocyte senescence by reducing SA-β-Gal activity and p16 expression while restoring nuclear lamin B1 levels and suppressing SASP. In a PD mouse model, AS-IV also protected against dopaminergic neuron loss and behavioral impairments, effects associated with a reduced accumulation of senescent astrocytes in the substantia nigra pars compacta [147].

### 4.3. Spices

Over the past decade, numerous studies have underscored various spices’ broad spectrum of anti-aging and anti-senescence properties. For instance, the primary bioactive compounds of black pepper, including piperine, chavicine, and sabinene, exhibit significant pharmacological potential. Notably, in vitro studies have shown that black pepper oil, which contains terpenoid compounds such as β-caryophyllene, limonene, β-pinene, and sabinene, has reduced the percentage of doxorubicin-induced senescent cells in CHO-K1 and NIH-3T3 cells [252]. Furthermore, curcumin, the primary component of Curcuma longa, has demonstrated a capacity to mitigate age-related deterioration by counteracting oxidative stress [253], modulating inflammatory pathways [254,255], promoting telomere elongation and telomerase activity [150], and influencing key metabolic regulators such as AMPK [256,257] and SIRTs [258,259]. Similarly, coriander seeds, which are rich in phenolic acids, coumarins, flavonoids, carotenoids, tocopherols, fatty acids, and sterols, have shown potential in reducing oxidative stress and cellular senescence, as evidenced by the decreased expression of senescence markers SA-β-Gal and p21 in the cardiac [260] and brain tissues [261] of obese rats.

Beyond their culinary roles, spices have emerged as promising agents for preventing or even counteracting neurodegenerative processes associated with aging. The neuroprotective effects of spices show promising therapeutic potential in PD as well. Curcumin has exhibited multiple protective mechanisms in PD, facilitated by its ability to cross the blood–brain barrier due to its lipophilic nature [262]. Its neuroprotective effects are attributed mainly to its potent antioxidant properties, surpassing conventional antioxidants such as vitamins C and E [263,264]. The ability of curcumin to donate hydrogen ions from its β-diketone moiety is believed to underlie its anti-ROS activity [265]. Notably, pre- or post-treatment administration of curcumin in 6-OHDA-lesioned rats reduced dopaminergic neuron loss [151], while MES cells treated with curcumin exhibited increased Cu-Zn superoxide dismutase expression and reduced intracellular ROS accumulation [152]. Moreover, curcumin was found to modulate inflammatory processes by decreasing the production of prostaglandins, glutamate, and pro-inflammatory cytokines in the hypothalamus, as well as reducing GFAP levels, a marker of astrocytic proliferation [266].

Similarly, piperine, the principal bioactive component of Piper nigrum (black pepper) has demonstrated neuroprotective effects in PD models. Yang et al. reported that piperine administration ameliorated MPTP-induced motor and cognitive deficits while preventing the loss of tyrosine hydroxylase-positive neurons in the substantia nigra [157]. Additionally, piperine reduced microglial activation, IL-1β expression, and oxidative stress and exhibited anti-apoptotic properties by modulating the Bcl-2/Bax ratio. Interestingly, piperine has been evaluated in combination with quercetin due to its well-documented ability to enhance the bioavailability of other compounds [267]. Combining quercetin and piperine improved MPTP-induced behavioral and neurochemical deficits while mitigating oxidative stress and inflammation in the striatum [268].

Emerging in vitro evidence further supports the beneficial role of cinnamon and its metabolites in PD. Cinnamaldehyde (10 μM) was shown to protect BE(2)-M17 human neuroblastoma cells from MPP+-induced toxicity by inhibiting autophagy [160]. Cinnamon extract (CEppt), with its main bioactive component cinnamaldehyde, has also shown protective effects against 6-OHDA-induced cytotoxicity by enhancing cell viability, reducing apoptosis, and decreasing ROS levels [161]. Furthermore, CEppt has been found to interfere with α-synuclein aggregation by stabilizing its soluble oligomeric form and promoting the disassembly of preformed aggregates [162].

In addition to their anti-inflammatory properties, these bioactive compounds exert antioxidative effects and inhibit acetylcholinesterase activity and Aβ aggregation in AD. Curcumin has demonstrated potent antioxidant activity in both in vitro and in vivo models [269,270,271]. Mechanistically, curcumin enhances the macrophage-mediated clearance of Aβ plaques [153], suppresses microglial proliferation [272], attenuates neuroinflammation by downregulating pro-inflammatory cytokines [273,274], and inhibits oxidative stress by preventing free radical formation and propagation [154,275]. Remarkably, in vitro studies suggest that curcumin reduces Aβ levels by modulating APP processing and downregulating BACE1 expression [155]. Additionally, curcumin exhibits a high binding affinity for Aβ aggregates, thereby preventing their formation both in vitro and in vivo [156].

In addition to Curcuma longa, Cinnamomum verum has demonstrated significant neuroprotective properties. Studies have shown that cinnamon has potent antioxidant effects, boosting the activity of endogenous antioxidant enzymes through various antioxidant biomolecules. These include cinnamic acid, which is widely reported, and some phenolic compounds, such as proanthocyanidins A and B and kaempferol [276,277], which also simultaneously exert anti-inflammatory effects [163,278]. Notably, CEppt effectively inhibits the formation of toxic Aβ oligomers and protects neuronal PC12 cells from Aβ-induced toxicity, eliminating tetrameric Aβ species in the brain. Moreover, oral administration of this extract in an aggressive AD transgenic mouse model significantly reduced 6 kDa Aβ oligomers, diminished plaque burden, and improved cognitive performance [164].

Piperine has been reported to exert neuroprotective effects [158]. In animal models, black pepper administration reduced cholinesterase activity and amyloid plaque formation in the brain [159]. Furthermore, piperine significantly attenuated lipid peroxidation and acetylcholinesterase activity while preserving neuronal density in adult male Wistar rats [158].

Similarly, cardamom oil treatment, constituted by 1,8-cineole, α-terpinyl acetate, limonene, linalyl acetate, and linalool, improved neurobehavioral parameters in male Wistar rats, inhibited acetylcholinesterase activity in the hippocampus and cortex, and enhanced antioxidant enzyme levels while reducing oxidative damage. Additionally, it increased BDNF levels and suppressed Aβ expression in the hippocampus and cortex [165].

Overall, accumulating evidence suggests that spices such as Curcuma longa, cinnamon, black pepper, and cardamom possess significant neuroprotective properties that may be exploited to prevent and treat neurodegenerative diseases, including AD and PD.

### 4.4. Dietary Fiber

Recent findings suggest that a high-fiber diet may protect against neurodegenerative disorders by supporting metabolism, modulating neuroinflammation, and regulating epigenetics. Unfortunately, although it displays several effects on the key mechanisms of cellular senescence, a direct link to senescence itself remains unclear. Dietary fiber, composed of non-digestible and non-absorbable carbohydrates, influences gut microbiota composition and short-chain fatty acid (SCFA) production, impacting brain function through the microbiota–gut–brain axis [279,280].

Shi et al. studied dietary fiber deficiency (FD) in mice, revealing alterations in hippocampal synaptic ultrastructure, the proteome, and microglial–neuroinflammation pathways, affecting cognition and dopamine cholinergic synapses [279].

Gut microbiome alterations have also been linked to PD, with decreased SCFAs, particularly butyrate, observed in PD patients [281]. Similarly, Matt et al. showed that butyrate administration and high-fiber diets reduced neuroinflammation in aged mice [166].

For AD, dietary fiber and SCFAs have shown benefits in cholesterol reduction, Aβ clearance, and neuroinflammation regulation, potentially mitigating Aβ deposition and brain hypometabolism [282,283,284,285].

Furthermore, in vitro studies found valeric, butyric, and propionic acids to interfere with neurotoxic Aβ aggregation [167], while in vivo, fiber influenced amyloid load in GPCR KO mice, suggesting a role in amyloid metabolism [168].

### 4.5. Probiotics and Prebiotics

Probiotics and prebiotics influence human health by modulating metabolic regulation, immune response, and neurological function via the gut microbiome [286,287,288,289,290]. Probiotics, particularly Lactobacilli species, have demonstrated benefits in aging by enhancing immunity and maintaining gut microbiota balance, potentially extending the healthspan [289,291,292,293]. Probiotics can regulate neuroinflammation and oxidative stress in neurodegenerative diseases, reducing the risk of disorders like PD and AD [169,170,171,294]. Furthermore, as previously mentioned, in synergy with polyphenols, probiotics can also function as hormetic nutrients modulating antioxidant and anti-inflammatory signaling pathways [54].

In PD, probiotics improve gut health, mitigate permeability, and reduce neuroinflammation [295]. Cellular studies show that probiotic treatment can shift cytokine production towards an anti-inflammatory profile [172], while in vivo studies suggest protection against dopaminergic neuron loss and neurotrophic factor depletion [173,174,175].

Similarly, specific probiotic strains in rat models can restore gut microbiota balance in AD, improve cognitive function, and mitigate pathological features such as Aβ deposition and oxidative stress [296,297]. However, the precise mechanisms remain unclear [176,177], and probiotics’ role as modulators of cellular senescence per se is only beginning to be understood.

Conversely, prebiotics, including fructo- and galacto-oligosaccharides, promote SCFA production and support cognitive function [298]. They modulate oxidative damage, enhance GLP-1 secretion, and potentially regulate brain glucose metabolism and CNS inflammation via GLP-1 receptors [299,300]. Prebiotics modulate gut microbiota composition in PD, reducing pro-inflammatory bacteria and increasing SCFA-producing taxa with promising neuroprotective effects [178,179]. Intriguingly, their combination with probiotics, via the consumption of fermented milk containing multiple probiotic strains and prebiotic fiber [301], appears particularly beneficial in PD patients, improving gut motility [301,302].

On the other hand, prebiotic supplementation in AD rodent models has shown improvements in neurotransmitter levels, cognitive function, and Aβ pathology, with chitosan oligosaccharides demonstrating neuroprotective properties in several studies [180].

Despite strong evidence supporting the role of probiotics and prebiotics in neuroinflammation and neurodegeneration, their direct impact on cellular senescence remains unclear. However, they have been shown to exert regulatory effects on oxidative stress, inflammation, and metabolism. Notably, they may modulate neuroinflammation, which is at least partially influenced by the presence of senescent cells. Further studies are needed to clarify this relationship and explore their potential in aging and neurodegenerative diseases.

### 4.6. Polyunsaturated Fatty Acids (PUFAs)

PUFAs are crucial in neuroprotection, presenting potential therapeutic implications for neurodegenerative diseases. Evidence supports their involvement in modulating inflammation, oxidative stress, and neurotoxicity. However, further research is required to investigate their direct action as senolytic or senostatic agents and their influence on the broader aging process [303]. PUFAs play a fundamental role in neurodevelopment, neurotransmission, and neuromodulation, with potential neuroprotective effects that include reducing neuroinflammation, mitigating neurotoxicity, promoting neural recovery, and preserving blood–brain barrier integrity [304].

Among PUFAs, omega-3 (n-3) and omega-6 (n-6) long-chain polyunsaturated fatty acids (LCPUFAs) are essential for brain function, constituting 30–35% of total brain fatty acids. Docosahexaenoic acid (DHA) and AA are the predominant LCPUFAs in phospholipids, playing key roles in synaptic integrity, plasticity, and cognitive function [305,306]. Neuroinflammation is a major contributor to age-related neurodegeneration, and n-3 LCPUFAs, particularly eicosapentaenoic acid (EPA) and DHA, exhibit anti-inflammatory properties by downregulating IL-6 and TNF-α while enhancing cognitive function [307]. Interestingly, higher brain DHA concentrations correlate with improved cognitive performance, likely due to its effects on membrane fluidity, neurotransmitter release, gene expression, neuroinflammation, and neuronal growth [308,309]. These fatty acids also possess antioxidant and anti-apoptotic effects, mitigating neurotoxicity in preclinical models [181,310].

Research on dietary fats is still emerging in PD, but observational studies suggest that LCPUFA intake may offer neuroprotection [182,311]. For example, a meta-analysis reported that higher LCPUFA consumption is associated with a reduced PD risk, while specific plasma fatty acid levels correlate with motor and non-motor symptoms [183]. Additionally, n-3 PUFAs have neuroprotective effects in MPTP-induced PD mice models, preventing neuronal loss and preserving striatal dopamine levels [181,184,312,313,314].

In AD, epidemiological studies and randomized controlled trials (RCTs) indicate that higher n-3 LCPUFA intake correlates with a lower incidence of cognitive impairment and dementia [185,309]. Other RCTs in individuals with mild-to-moderate AD have reported cognitive improvements following supplementation [186,315]. Animal studies suggest that DHA reduces amyloid accumulation, tau pathology, and synaptic dysfunction, with several independent reports confirming reduced Aβ levels in APP transgenic models following DHA-enriched diets [187,188,189,190,316].

## 5. Conclusions and Future Perspectives

This review highlights the important role of nutraceuticals and functional foods in reducing aging and neurodegenerative diseases by modulating cellular senescence and its related aspects. It discusses how these natural bioactive compounds possess potent antioxidant, anti-inflammatory, and epigenetic properties that can impact essential cellular pathways associated with aging and the onset of neurodegenerative diseases. We specifically emphasize the importance of polyphenols, vitamins, and spices as nutritional senotherapeutic agents in scavenging ROS, reducing the secretion of inflammatory SASP factors, and modulating gene expression, alongside other characteristics related to cell senescence.

Collectively, these actions contribute to alleviating the cellular damage involved in both aging and the onset of neurodegenerative disorders, such as AD and PD.

Even though this review highlights substantial evidence supporting the nutraceuticals’ beneficial effects on cellular senescence processes, such as improving mitochondrial function, reducing oxidative stress, and modulating inflammatory responses, direct evidence demonstrating a senolytic effect is still limited.

Most existing studies have primarily focused on elucidating the mechanisms through which these compounds influence senescence-associated characteristics rather than proving a direct reduction in the number of senescent cells. This emerging and relatively new field requires further research to explore these correlations in more detail and to understand the potential benefits of introducing nutraceuticals into preventive strategies. Such interventions may offer a promising approach to extending healthspan by targeting the underlying mechanisms of cellular senescence, although current research is still in its infancy. Ultimately, this review suggests that incorporating nutraceuticals into comprehensive dietary interventions may help reduce the risk of neurodegenerative diseases. However, the scarcity of clinical data raises questions about their effectiveness, especially considering the emerging hormetic properties of specific nutraceuticals. Last but not least is the issue of the doses to be used in vivo to achieve an effect, considering aspects of absorption, interactions, therapies, and individual characteristics that could influence functionality. Numerous questions remain unresolved regarding the application of nutraceuticals as senotherapeutics, but there exists a pressing necessity to identify anti-aging strategies that promote active longevity while minimizing disability and functional dependence.

Future research should overcome the current translational gap by prioritizing mechanistic studies utilizing transcriptomics, proteomics, and metabolomics to elucidate whether nutraceuticals exhibit senomorphic or senolytic properties and affect the corresponding pathways involved. Experimental models mimicking the brain’s cellular complexity, such as co-cultures and 3D organoids, should be employed to better capture the pathophysiology and the impact of nutraceuticals on brain senescence and neuroinflammation. In vivo studies using animal models of Alzheimer’s and Parkinson’s diseases are also necessary to evaluate senescence biomarkers, including pro-inflammatory cytokines, genetic markers, and cognitive outcomes.

Identifying characteristic proteins associated with senescence-related phenotypes and cataloguing potential senescence biomarkers is imperative. This work will aid in evaluating the burden of senescence, the stimuli that trigger this process, and the tissue origins of senescent cells in vivo. Such information could prove invaluable in assessing the therapeutic benefits of nutraceuticals in living organisms.

Moreover, enhancing the bioavailability of nutraceuticals through novel delivery systems and investigating their effects within comprehensive dietary contexts, such as the Mediterranean diet (MedDiet), will increase their translational potential. Clinically, pilot trials in frail or cognitively impaired older adults, supported by validated senescence biomarkers, could provide early insights into efficacy. Lastly, integration with genetic and epigenetic studies should be pursued to assess individual responses to nutraceuticals and develop highly personalized functional foods tailored to specific diet patterns.

## 6. Limitations

This review provides a comprehensive overview of the current literature on the role of nutraceuticals and functional foods in modulating cellular senescence and their potential implications for neurodegenerative diseases. Nonetheless, several limitations warrant consideration. Most available evidence stems from in vitro or animal studies, with limited clinical validation. Despite promising preclinical data, the clinical utility remains uncertain due to poor bioavailability, a short half-life, and inter-individual variability, which restricts translational relevance. Additionally, hormetic effects add a layer of complexity, as beneficial effects depend on dose optimization, a factor rarely addressed. Indeed, an additional challenge lies in determining effective in vivo dosing, since absorption, metabolism, and tissue distribution rarely correlate linearly with the administered dose. Additionally, age-related changes, causing reduced renal and hepatic clearance, an increased volume of distribution for lipophilic drugs, and prolonged elimination half-life, alongside heightened pharmacodynamic sensitivity, further complicate dose selection [317]. As a result, dosing must consider the target medical condition, patient-specific factors, and health status. The safety profiles of the reviewed molecules in humans are primarily still being defined. However, a safe dosage in humans has been gauged for some natural compounds, such as resveratrol (500 mg) [318], Ginkgo Biloba extract (120 mg) [319], and curcumin (1–6 g a day for 4–7 weeks) [320]. Some compounds discussed in this review exert pleiotropic effects on multiple biological pathways, challenging the attribution of their benefits to senescence-related mechanisms. Variability in compound purity, bioavailability, and dosing further complicates comparisons across studies in terms of robustness and significance. Collectively, these factors highlight the need for more rigorous, standardized, and clinically focused research.

## Figures and Tables

**Table 1:** Overview of nutraceutical compounds studied in the context of aging and neurodegenerative diseases. Experimental models, effects (mechanisms of action), observations, and corresponding references are reported for each compound. Abbreviations: dUCH: ubiquitin C-terminal hydrolase; LTP: Long-Term Potentiation; LTD: Long-Term Depression; PINK1: PTEN-induced kinase 1; MMP+: 1-Methyl-4-phenylpyridinium; MPTP: 1-Methyl-4-phenyl-1,2,3,6-tetrahydropyridine; RBC: red blood cells; t-BHP: tert-Butyl hydroperoxide; MDA: malondialdehyde; OPC: Oligodendrocyte Precursor Cells; GPx: Glutathione Peroxidase; 6-OHDA: 6-Hydroxydopamine; TBARS: Thiobarbituric Acid Reactive Substances; DA-D2: Dopamine D2 receptor; SAMP8: senescence-accelerated prone 8; hTERT: human Telomerase Reverse Transcriptase; TH: Tyrosine Hydroxylase; ΔΨm: Mitochondrial membrane potential; PBMC: Peripheral Blood Mononuclear Cells; swAPP: Swedish Amyloid Precursor Protein; AChE: Acetylcholinesterase; SCFA: Short-Chain Fatty Acids; MAO B: Monoamine Oxidase B; TTR: Transthyretin; DHA: Docosahexaenoic Acid; AA: arachidonic acid; hNT: human Neural Tissue; HO-1: Heme Oxygenase-1; DI TNC1: Rat type 1 astrocytes; H 19–7: rat hippocampal neurons; QR: Quinone reductase; GSTs: Glutathione S-transferase; N2a: Mouse neuroblastoma-derived cells; ADAM(10): metalloproteinase; ALDH1A1: Aldehyde dehydrogenase 1A1; SAM: S-adenosylmethionine; SAH: S-adenosylhomocysteine; MMSE: Mini–Mental State Examination; Ceppt: cinnamon extract; AS-IV: Astragaloside IV.

Nutraceuticals	Study Models	Effects	Observations	References
**Antioxidant** **vitamins**				
*Vitamin C*	Albino mice	Improves memory	↓ Acetylcholinesterase activity	[107]
			
	Drosophila dUCH Drosophila DJ-1β mutant (PD models)	Neuroprotective	↓ Dopaminergic neuron loss	[108] [109]
			
	SH-SY5Y cells (Aβ_25-35_ -treated)	Protects cells from Aβ_25-35_-mediated apoptosis	↓ Basal Aβ secretion	[110]
			
	Wistar rats Aβ- or artificial cerebrospinal fluid-injected (AD models)	↓ Oxidative stress ↓ Neuroinflammation	↓ Lipid peroxidation products ↓ IL-1β, IL-6, and TNFα	[111]
			
	Hs68 human dermal fibroblasts, H_2_O_2_-treated Middle-aged hairless mice, LPS-treated hAPCs	Prevents cellular senescence	↓ Hyperactivation of PI3K/AKT ↓ p53/p21 ↓pRB/p16 ↑ E2F1/E2F2 ↓ mTOR ↑ FoxO3a ↑ SIRT1	[112] [113]
				
*Vitamin E*	Questionnaire-based case–control study (healthy and PD patients) Brain slice of PINK1^−^/^−^ mice	Reduces PD occurrence Reverses impaired synaptic plasticity	Restored LTD and LTP	[114]
				
	Drosophila DJ-1β mutant (PD model)	↓ Oxidative stress ↑ Lifespan	↑ Catalase activity ↓ SOD	[109]
				
	SH-SY5Y cells treated with MPP+, MG132, and thapsigargin (PD model) Mice, MPTP-treated (PD model)	Neuroprotective against PD-related toxicities Antioxidant effects ↑ Motor function	Activation of ERβ/PI3K/Akt pathway	[115] [116]
				
	C57 black mice, MPTP-treated (PD model)	Prevent neuronal loss in substantia nigra	↓ Striatal dopamine loss	[117]
				
	Cross-sectional study (>40 years old)	Reduced risk of PD	-	[118]
				
	Primary rat embryonic hippocampal neurons, Aβ_1-42_-treated (AD model)	↓ Oxidative stress	Prevents Aβ_1-42_-induced neuronal protein oxidation Free-radical scavenger	[119]
				
	IL-1β-stimulated A549 cells LPS-stimulated RAW264.7 macrophages (inflammatory diseases model)	↓ Inflammation	↓ PGE_2_ COX_2_ inhibition	[120] [121]
				
	HUVECs Human primary dermal fibroblasts (replicative senescence) Human primary skin fibroblasts from young and aged subjects	Delays senescence	↓ Number of senescent cells ↓ p21	[122] [123]
*Vitamin A*	HEK293 cells	Cytoprotection	Inhibits Aβ oligomer formation	[124]
	Cortical neurons from embryonic mice, Aβ_1-40_- and Aβ_1-42_-treated 129S2/SvHsd and Tg2576 mice (AD models)	Neuroprotective	Inhibits Aβ oligomer formation ↑ Disintegrin ↑ADAM (10)	[125]
	SH-SY5Y cells	Hormetic effect ↓ Oxidative stress	↑ TH ↑ Akt and ERK1/2 phosphorylation	[126]
				
	Postnatal and adult Aldh1a1 knockout mice	↓ Dyskinesia	↑ MOR1	[127]
				
*Vitamin B*	AD patients	Cognitive improvement ↓ Neuroinflammation	↑ MMSE ↑ SAM/SAH ↓ Aβ_1-40_, PS1, and TNFα ↓ Blood homocysteine	[128] [129]
				
	SAMP8 mice Astrocytes from mice (Aging models)	↓ Neurodegeneration	↑ Telomerase activity ↓ Astrocitosis ↓ Apoptosis	[130]
	Gibco Human Astrocytes Vitamin-B12-deficient	↓ Senescence	↓ SA-β-Gal, p16, p21	[131]
**Polyphenols, Terpenes, and Terpenoids**				
*Quercetin*	WI-38 fibroblasts (Doxo-treated)	Prevents cellular senescence ↓ Senescent fibroblast pro-tumor effects	↑ SOD1 and SOD2	[132]
			
	WI-38 fibroblasts	Senolytic effect	↓ Autophagy ↑ ER stress	[133]
			
	Human RBC cells, *t*-BHP-treated (oxidative stress model)	↓ Deleterious effects of oxidative stress in erythrocytes	↓ MDA ↑ GSH ↑ Membrane-SH Group	[134]
*Quercetin + Dasatinib*	Aβ_1-42_-induced senescent OPC cells APP/PS1 transgenic mice (AD model)	Senolysis of senescent OPCs ↓ Neuroinflammation ↑ Cognitive function	Inflammation, senescence, Aβ pathology	[135]
				
*Ginkgolides and* *bilobalide*	C57BL/6J mice, MPTP-treated (PD model)	Protect against nigrostriatal dopaminergic neurotoxicity ↑ Locomotion activity ↓ Oxidative stress	↓ Lipid peroxidation ↓ Mn-SOD ↑ GPx activity ↑ Glutathione reductase Inhibitory effect of brain	[136] [137]
				
*Resveratrol*	Wistar rats, 6-OHDA-treated (PD model)	↑ Antioxidant status ↓ Dopamine loss	↓ TBARS ↑ GSH, TH, Na^+^/K^+^-ATPase activity ↓ DA-D2 receptor binding ↓ PLA2 and COX-2	[138]
			
	SK-N-BE cells, 6-OHDA-, Aβ_1-42_-, and α-sin-treated (oxidative stress, PD, and AD models)	Neuroprotection ↓ Oxidative stress	Activates SIRT1 ↑ Autophagy	[139]
			
			
*Oleuropein*	SH-SY5Y and OLN-93 cells, α-synuclein-treated (PD models)	Stabilizes α-synuclein monomers Prevents pathological aggregation ↓ Cytotoxicity ↓ Oxidative stress	↑ α-Synuclein proteolysis ↓ α-Synuclein interaction with cell membrane ↓ LDH release	[140] [141]
				
*Fisetin*	Aged SAMP8 mice (AD model)	Prevents cognitive and locomotor deficits with age ↓ Neuroinflammation	↓ SAPK/JNK Metabolic alteration	[142]
				
*Limonene*	Adult Mediterranean fruit flies (aging model)	↑ Lifespan	Hormetic effect	[143]
				
*Ginsenoside F1*	Human astroglioma CRT and U373-MG cells (20 g/L D-galactose-induced senescence)	Suppresses the SASP ↓ Astrocyte-derived neuroinflammation	↓ p38MAPK-dependent Nf-κB	[144]
				
	Mouse sw APP N2a cells (AD model)	Reduces Aβ_1-40_ and Aβ_1-42_ formation	↑ PPARγ ↓ BACE1	[145]
				
*Artemisin*	LPS-activated RAW 264.7 macrophages	↓ Inflammation	↓ AChE	[146]
				
*AS-IV*	Replicative-induced and LPS/MPP^+^-induced senescent mouse astrocytes Mice, MPTP-treated (PD models)	↓ Inflammation Neuroprotection ↑ Longevity ↓ Dopaminergic neuron loss	Attenuates senescence and SASP ↑ TH ↑ Autophagy	[147]
**Spices**				
*Curcumin*	Astrocytes DI TNC1 and neurons H 19–7 from rats	Cytoprotection against oxidative stress	↑ HO-1 and Nrf2 ↑ QR and GSTs	[148]
				
	Human AD and Tg2576 mouse brain sections swAPP Tg2576 transgenic mice (AD model) Differentiated SH-SY5Y cells, Aβ-treated (AD model)	Blocks Aβ aggregation Prevents Aβ cytotoxicity	Labels amyloid plaques in the brain Induces disaggregation of pre-aggregated Aβ	[149]
				
	HEK293T (hTERT-transfected)	↑ Telomere elongation	↑ Telomerase activity	[150]
			
	Sprague Dawley rats, 6-OHDA-treated (PD model)	Neuroprotective	↓ Loss of TH-positive cells and DA content	[151]
			
	MES23.5 cells, 6-OHDA-treated (PD model)	Protects from neurotoxicity	Restores ΔΨm ↑ Cu-Zn SOD ↓ ROS ↓ NF-κB activation	[152]
			
	PBMC from healthy and AD patients	↑ Aβ clearance	↑ AD macrophage-mediated Aβ phagocytosis	[153]
			
	PC12 rat cells and HUVECs, Aβ-treated (AD model)	Protects from Aβ_1-42_ insult	↑ Antioxidant pathway	[154]
			
	swAPP HEK293 cells (AD model)	↓ Aβ_1-42_ production	↓ APP protein expression	[155]
			
	In vitro *(cell-free)*	Inhibits aggregation	Inhibits Aβ_1-40_ and Aβ_1-42_ fibril formation and extension	[156]
				
*Piperine*	C57BL/6 mice, MPTP-treated (PD model)	↓ MPTP-induced deficits in motor coordination and cognitive functioning Prevents decrease in TH-positive cells	↑ Bcl2/Bax ratio ↓ Oxidative stress ↓ Microglia activation ↓ IL-1β	[157]
			
	Wistar rats, AF64A-injected (AD model)	Improves memory impairment and neurodegeneration in hippocampus	↓ Lipid peroxidation ↓ AChE activity	[158]
			
	Albino rats, aluminum-chloride-injected (AD model)	Prevents neurodegeneration ↑ Memory	↓ AChE activity	[159]
				
*Cinnamaldehyde* *and CEppt*	BE(2)-M17 cells	Prevents neuronal death in the substantia nigra	Autophagy	[160]
			
	PC12 cells (6-OHDA-treated)	Protective against 6-OHDA-induced cytotoxicity	↑ Survivin ↓ Cyt-c Oxidative stress, apoptosis	[161]
				
	Drosophila mutated for A53T α-synuclein in the brain (model of PD)	Neuroprotective	Interferes with α-synuclein aggregation Promotes disassembly of performed aggregates	[162]
			
	THP-1 monocytes, LPS-treated (inflammatory model)	↓ Inflammation	↓ Akt and IκBα phosphorylation	[163]
	PC12 cells, Aβ-treated Drosophila, Aβ_42_-transfected 5XFAD mice (AD models)	Inhibits formation of toxic Aβ oligomers Improves cognitive behavior Ameliorates locomotion defects	Prevents Aβ cytotoxicity Aβ aggregation ↓ Aβ plaques	[164]
				
*Cardamom oil*	Wistar rats, aluminum-chloride-injected (AD model)	Improves behavioral parameters ↓ Oxidative stress ↓Neuronal damage ↓ Aβ plaques	↓ AChE activity	[165]
**Dietary Fiber**				
	Adult and aged Balb/c mice	↓ Inflammatory infiltrate	↑ Butyrate gut microbiota ↑ SCFA production	[166]
			
	In vitro *(cell-free)*	Inhibits Aβ_1-40_ and Aβ_1-42_ aggregation	Protein interaction	[167]
			
	5xFAD mice (AD model)	Delays cognitive decline ↑ Cognitive function ↑ Memory	Alters microglial transcriptome Alters T-cell profile in the brain	[168]
**Probiotics**				
	Accelerated-aging C57BL/6 mice	↓ Inflammation ↑ Neurotrophic factor ↑ Memory	↓ p16, NF-κB, iNOS, and COX-[2]	[169]
	C. elegans, H_2_O_2_-treated HT-29 cells stimulated with proinflammatory cytokines	↑ Lifespan Anti-inflammatory ↓ Oxidative stress	Modulation of DAF[2]/DAF-[16] pathway	[170]
				
	D-galactose-induced oxidative stress, ICR mice	↑ Antioxidant status ↓ Liver damage ↓ Lipid peroxidation	↑ Nrf[2]/Keap[1] ↑ SOD ↑ GPx	[171]
				
	PBMCs from healthy and PD patients	↓ Inflammation ↓ Oxidative stress ↑ Anti-inflammation	Restore membrane integrity ↓ Pathogenic bacteria	[172]
				
	SH-SY5Y cells (dopaminergic phenotype) C57BL/6 mice, 6-OHDA-treated (PD models)	↑ Synaptic plasticity ↑ Neuroprotection ↓ Neuroinflammation	↑ PI[3]K/Akt, NF-κB, and PPARγ ↓ JNK/ERK	[173]
				
	C57BL/6 mice, MPTP- and rotenone-treated (PD models)	↓ Motor deficits ↓ Neuroinflammation ↓ Oxidative stress Neuroprotective	↑ Neurotrophic factors and butyrate level ↓ Glial reactivity Antioxidant enzymes Gut microbiota ↓ Dopaminergic neuronal death ↓ MAO B	[174] [175]
				
	Aged Fischer 344 rats	↓ Inflammation Ameliorate age-dependent memory impairment	↓ NF-κB ↓ p[16], COX-[2], and iNOS in the hippocampus	[176]
				
	ddY-mice, Aβ_1-42_-injected (AD model)	↓ Inflammation Prevent cognitive dysfunction	↓ Immune-reactive-related genes	[177]
**Prebiotics**				
	Healthy and PD patients	↓ Inflammation ↓ Neurodegeneration ↓ Non-motor symptoms	↑ Beneficial metabolites Change microbiota	[178] [179]
			
	D-galactose- and Aβ_1-42_-induced deficient Sprague Dawley rats (AD model)	↓ Oxidative stress ↓ Inflammation ↑ Learning and memory abilities	↓ Tau and Aβ_1-42_ expression Modulate microbiota–gut–brain axis	[180]
**PUFAs**				
	C57BL/6 mice, MPTP-treated (PD model)	Neuroprotective	Prevent decrease in TH-labeled nigral cells Protect from dopamine decrease	[181]
				
	Human subjects (>55 years old) PD patients	Lower the risk of PD	Modify the association of PD with paraquat and rotenone	[182] [183]
				
	C57BL/6 mice, MPTP-treated (PD model)	Neuroprotective	↑ BDNF	[184]
				
	Wistar rats, Aβ-treated (AD model)	Neuroprotective	↓ ROS, NOX1, MAO ↑ NOX2, DOS1, serotonine Prevent the ↓ of IL-10	[185]
				
	AD patients	Reduce Aβ in the brain	↑ TTR that binds and reduces Aβ	[186]
				
	Aged transgenic Tg2576 mice (AD model)	Neuroprotective	↑ PI3K/Akt ↓ BAD	[187]
				
	Old 3xTg AD mice	Ameliorate cognitive performance	Ameliorate DHA/AA balance	[188]
				
	5XFAD mice (AD model) Mouse astrocytes and microglia, LPS-stimulated	↓ Inflammation Ameliorate cognitive deficits	↓ Soluble form of Aβ ↑ Abca1 and ApoE gene expression	[189]
				
	swAPP/PS1ΔE9 tg mice hNT neuronal cultures (AD models)	Prevent amyloid toxicity	↓ Plaque ↑ Drebrin in hippocampus	[190]

## Abbreviations

The following abbreviations are used in this manuscript:

αT	α-tocopherol
αTP	α-tocopheryl phosphate
Aβ	Amyloid-β peptide
AA	Arachidonic acid
AAC	Ascorbic acid
AD	Alzheimer’s disease
ARDs	Age-related diseases
BACE1	β-secretase 1
CEppt	Cinnamon extract
CNS	Central nervous system
CR	Caloric restriction
D+Q	Dasatinib plus quercetin
DHA	Docosahexaenoic acid
DOPA	Dihydroxyphenylalanine
EPA	Eicosapentaenoic acid
FD	Fiber deficiency
GLP-1	Glucagon-like peptide 1
GSH	Glutathione
GSs	Geriatric syndromes
IL	Interleukin
LCPUFAs	Long-chain polyunsaturated fatty acids
MAO-B	Monoamine Oxidase B
MDA	Malondialdehyde
MedDiet	Mediterranean diet
MPTP	1-methyl-4-phenyl-1,2,3,6-tetrahydropyridine
NFTs	Neurofibrillary tangles
PD	Parkinson’s disease
PUFAs	Polyunsaturated fatty acids
ROS	Reactive oxygen species
SASP	Senescence-associated secretory phenotype
SA-β-gal	Senescence-associated β-Galactosidase
SAHFs	Senescence-associated heterochromatic foci
SCFAs	Short-chain fatty acids
SIRT	Sirtuin
